# Structural and functional characteristics of soil microbial communities in response to different ecological risk levels of heavy metals

**DOI:** 10.3389/fmicb.2022.1072389

**Published:** 2022-12-08

**Authors:** Dale Li, Jianwen Chen, Xiujuan Zhang, Wei Shi, Junjian Li

**Affiliations:** Institute of Loess Plateau, Shanxi University, Taiyuan, China

**Keywords:** heavy metals, ecological risk level, microbial function, microbial community assembly, microbial structure

## Abstract

**Objective:**

The potential ecological risk index (RI) is the most commonly used method to assess heavy metals (HMs) contamination in soils. However, studies have focused on the response of soil microorganisms to different concentrations, whereas little is known about the responses of the microbial community structures and functions to HMs at different RI levels.

**Methods:**

Here, we conducted soil microcosms with low (L), medium (M) and high (H) RI levels, depending on the Pb and Cd concentrations, were conducted. The original soil was used as the control (CK). High-throughput sequencing, qPCR, and Biolog plate approaches were applied to investigate the microbial community structures, abundance, diversity, metabolic capacity, functional genes, and community assembly processes.

**Result:**

The abundance and alpha diversity indices for the bacteria at different RI levels were significantly lower than those of the CK. Meanwhile, the abundance and ACE index for the fungi increased significantly with RI levels. Acidobacteria, Basidiomycota and Planctomycetes were enriched as the RI level increased. Keystone taxa and co-occurrence pattern analysis showed that rare taxa play a vital role in the stability and function of the microbial community at different RI levels. Network analysis indicates that not only did the complexity and vulnerability of microbial community decrease as risk levels increased, but that the lowest number of keystone taxa was found at the H level. However, the microbial community showed enhanced intraspecific cooperation to adapt to the HMs stress. The Biolog plate data suggested that the average well color development (AWCD) reduced significantly with RI levels in bacteria, whereas the fungal AWCD was dramatically reduced only at the H level. The functional diversity indices and gene abundance for the microorganisms at the H level were significantly lower than those the CK. In addition, microbial community assembly tended to be more stochastic with an increase in RI levels.

**Conclusion:**

Our results provide new insight into the ecological impacts of HMs on the soil microbiome at different risk levels, and will aid in future risk assessments for Pb and Cd contamination.

## Introduction

Healthy soils can sustainably support the survival of plants, animals, and humans. They also provide a range of important ecosystem services (Lehmann et al., 2020). A main threat to healthy soil is heavy metals (HMs) contamination from human activities, which include over-mining, smelting, pesticide spraying, and indiscriminate dumping of waste rock and slag (Xiang et al., 2021; Zerizghi et al., 2022). Excessive accumulation of Pb and Cd in soil can contribute to growth inhibition and metabolic disorders in plants, which result in a reduction in crop yield and quality (Shahid et al., 2012; Wei et al., 2018). Low concentrations of Pb and Cd may also be harmful to organisms at different nutritional levels in the food chain (Vries et al., 2007). Many diseases affecting humans are associated with HMs exposure. Cd can cause emphysema, osteoporosis, and cancer in humans (Chen et al., 2018). Meanwhile, Pb exposure can cause neurological changes and loss of function (Huang et al., 2018). Scientific assessment of the ecological risks of Pb and Cd contamination is essential to support sustainable agricultural development and limit risks to human health.

The potential ecological risk index (*RI*) is an approach commonly used to evaluate HMs pollution in soils (Cui et al., 2022; Wei et al., 2022). As a key parameter of the *RI*, the applicability of toxicity factors (TF) in ecological risk assessments for microorganisms has been poorly studied. Our previous research has shown that the TF of Pb and Cd was more suited for evaluating the impacts of HMs on microbial abundance and diversity than their concentrations (Li et al., 2022). However, to date, the main research focus has been on the impacts of different concentrations of HMs on microbial communities (Fan et al., 2021; Yang J. et al., 2022). Microbial responses to different *RI* levels have not yet been adequately assessed. This limits our ability to accurately evaluate the ecological risks of HMs contamination.

Soil microorganisms play an important role in various biogeochemical processes including decomposition of soil organic matter, humification, cycling of soil carbon and nitrogen, and ecosystem function (Xing et al., 2022; Yang Y. et al., 2022). Microbial diversity tends to be enhanced by HMs stimulation at low concentrations, whereas the stress impacts of HMs at high concentrations may result in reduced microbial diversity and abundance (Ma et al., 2021; Qi R. et al., 2022). Microbial communities comprise few abundant species and numerous rare species (Saunders et al., 2016). Both abundant and rare taxa have been shown to respond differently to HMs stress (Wang M. et al., 2022). Microbial interactions are essential for survival of microorganisms in soils polluted with HMs (Chun et al., 2021; Sun et al., 2022). Qi Q. et al. (2022) highlighted that the complexity of microbial networks increases with HMs concentration. Furthermore, community assembly is critical in assessing the impacts of environmental contaminants on soil microbial communities (Sun et al., 2021; Liu H. et al., 2022). Microbial diversity, activity, stability, and assembly have been extensively documented as important indicators for evaluating the impact of different concentrations of HMs on microorganisms (Fierer et al., 2021; Ding et al., 2022; Nikitin et al., 2022). Despite this, how microbial indicators and community assembly change as *RI* levels increases remains unclear.

HMs contamination can affect soil ecosystem functions (Jiang et al., 2019). However, different microbial communities can have identical ecosystem functions (Yang et al., 2021). This means that only focusing on changes in microbial communities is insufficient for identifying soil functions. Evaluating the ability of microorganisms to metabolize carbon sources immediately reflects the metabolic demands of microbial communities exposed to HMs (Borgulat et al., 2021). Soil microorganisms can also promote carbon, nitrogen, phosphorus, and sulfur cycles (Tian et al., 2021; Wang X. et al., 2022). Zhao et al. (2021) found that Cd contamination stimulated nitrogen capture by microbial communities from initially dissolved organic nitrogen to later refractory organic nitrogen. Xu et al. (2021) showed that an increase in HMs concentrations considerably increased carbon limitation. Therefore, it is worth exploring how these functions change with *RI* levels.

In this study, we conducted soil microcosm experiments using Pb and Cd at different *RI* levels, with the aim of investigating: (1) responses of microbial community diversity and interactions, (2) changes in microbial community metabolism and function, and (3) microbial community assembly mechanisms. The findings of this study will help to elucidate the responses of microbial indicators at different *RI* levels and inform future scientific assessments and ecological remediation practices for Pb and Cd contamination.

## Materials and methods

### Soil microcosm incubation

Soil samples were collected from the Tianlong Mountain Nature Reserve (37°42′N, 112°27′E) in Shanxi Province, China. The soil chemistry and HMs contents are described in Supplementary Table S1. The specific steps and culture conditions for construction of the soil microcosms have been described by Li et al. (2022). According to the *RI* assessment criteria and *RI* calculation formula (Supplementary Tables S2, S3), we conducted soil microcosms for low (L, *RI* = 100), moderate (M, *RI* = 200), and high (H, *RI* = 400) *RI* levels. The original soil samples were used as the control (CK). The three *RI* levels were reached by adding different amounts of Pb (CH_3_COO)_2_·3H_2_O and CdCl_2_·2.5H_2_O. The specific additions were based on previous research (Supplementary Table S4; Li et al., 2022). Different treatments at the same *RI* level were treated as replicates. There were five treatments for L level and six treatments each for M and H levels, respectively. The soil samples were collected on day 45 of each treatment prior to further analysis.

### DNA extraction, qPCR, and sequencing

The E.Z.N.A® Soil DNA Kit (Omega BioTek, Norcross, GA, United States) was used to extract the total DNA of the microorganisms from the soil samples (Franco Ortega et al., 2018). qPCR was used to quantify 16S rRNA and ITS genes to evaluate the abundances of bacteria and fungi, respectively. The primer sequences used for high-throughput sequencing were the same as those used for the qPCR analysis (Supplementary Table S5). The 16S rRNA and ITS genes were sequenced with the Illumina MiSeq platform using the PE300 (2 × 300) by Majorbio Bio-Pharm Technology Co. Ltd. (Shanghai, China). The raw sequences were analyzed according to the method described by Pan et al. (2020). Alpha diversity indices, including ACE, Shannon, and Phylogenetic Diversity (PD), were determined using the Majorbio platform.^1^ The diversity indices here refer to the genetic diversity indices.

### Metabolic profile evaluation

The Biolog® ECO and FF plate (Biolog Inc., Hayward, CA, United States) test methods were applied to assess the functional metabolism of the bacterial and fungal communities, respectively (Gryta et al., 2020). Biolog ECO plates are 96-well microplates that contain 31 substrates in triplicate and three control wells without a substrate (Feigl et al., 2017). The 31 carbon sources were classified into six substrate groups, namely carbohydrates (12), amino acids (6), polymers (4), carboxylic acids (5), amines (2), and phenolic compounds (2) (Wasak et al., 2020). Biolog FF plates contain 95 carbon substrates from eight biochemical groups, namely carbohydrates (29), carboxylic acids (20), amino acids (10), glycosides (4), polymers (5), polyols (10), amines and amides (9), and miscellaneous (8) (Wasak et al., 2020). Each plate also contains water as the negative control. The measurements were made as described by Ge et al. (2018). Average well color development (AWCD) was used to examine the carbon metabolic activity of the microbial communities (Ge et al., 2018). The Shannon–Wiener diversity index (*H′*), Simpson diversity index (*D*), and McIntosh index (*U*) were calculated as functional diversity indices, and all the formulas used are shown in Supplementary Table S3.

### High-throughput quantitative PCR of functional genes

A total of 72 functional genes associated with C, N, P, and S were measured using QMEC based on the high-throughput quantitative PCR (HT-qPCR) technique (Guangdong Mega Gene Technology Co., Ltd.). Target genes with amplification efficiencies greater than the range (1.8–2.2) were eliminated (Jia et al., 2022). The detection limit was set at a threshold cycle (CT) of 31. Each qPCR response was performed in triplicate; all primer pairs are summarized in Supplementary Table S5.

### Statistical analysis

One-way analysis of variance (ANOVA) was used to determine the variability in bacterial/fungal abundance, genetic diversity, functional diversity, AWCD, and functional gene abundance among the different *RI* levels. According to the Bray–Curtis dissimilarity metric (Katz et al., 2022), the PCoA was used to investigate the differences in bacterial and fungal community structures among all the samples. The statistically significant differences among all the samples were determined using PERMANOVA with the *vegan* package in R (Stefanowicz et al., 2022). We divided the microbial taxa into abundant (relative abundance > 1%) and rare (relative abundance < 1%) taxa for community structure analysis (Yi et al., 2022).

The online Molecular Ecological Network Analysis (MENA) pipeline^2^ used co-occurrence networks to assess interactions between bacterial and fungal communities (Deng et al., 2016). Based on the random matrix theory and Pearson correlation coefficient, we conducted the network analysis by selecting the operational taxonomic unit (OTUs) of bacteria and fungi with relative abundances > 0.01%. The MENA interface was used to determine the network topological indices, including nodes, links, modularity (M), average clustering coefficient (avgCC), and average path distance (GD). According to the approach of Deng et al. (2012), keystone taxa were distinguished based on within-module connectivity (Zi) and among-module connectivity (Pi). The stability of the microbial networks was assessed by calculating the robustness and vulnerability of each (Yuan et al., 2021). Robustness was defined as the random removal of 50% of the nodes, and the results were based on 100 iterations of the simulation. Vulnerability is represented by the maximum vulnerability of the nodes in the network. ANOVA was used to determine variability in the robustness and vulnerability among different *RI* levels. A co-occurrence network of bacterial communities and functional genes was generated using data from all the samples. All the networks were visualized using Gephi 0.9.2.

To identify the assembly mechanisms of the microbial communities at different *RI* levels, R scripts were performed to execute the neutral community model (NCM) and determine the modified normalized stochasticity ratio (MST). In NCM, R^2^ and m represent the overall fit to the neutral model and estimated migration rate, respectively (Sloan et al., 2006). The MST approach enables the relative importance of deterministic and stochastic processes to be quantified. This means that the community assembly is dominated by deterministic processes for MST values < 0.5 and stochastic processes for MST values > 0.5 (Ning et al., 2019). The results of both methods were visualized using R (v 4.0.2).

## Results

### Microbial community diversity and composition

**Figure 1:**
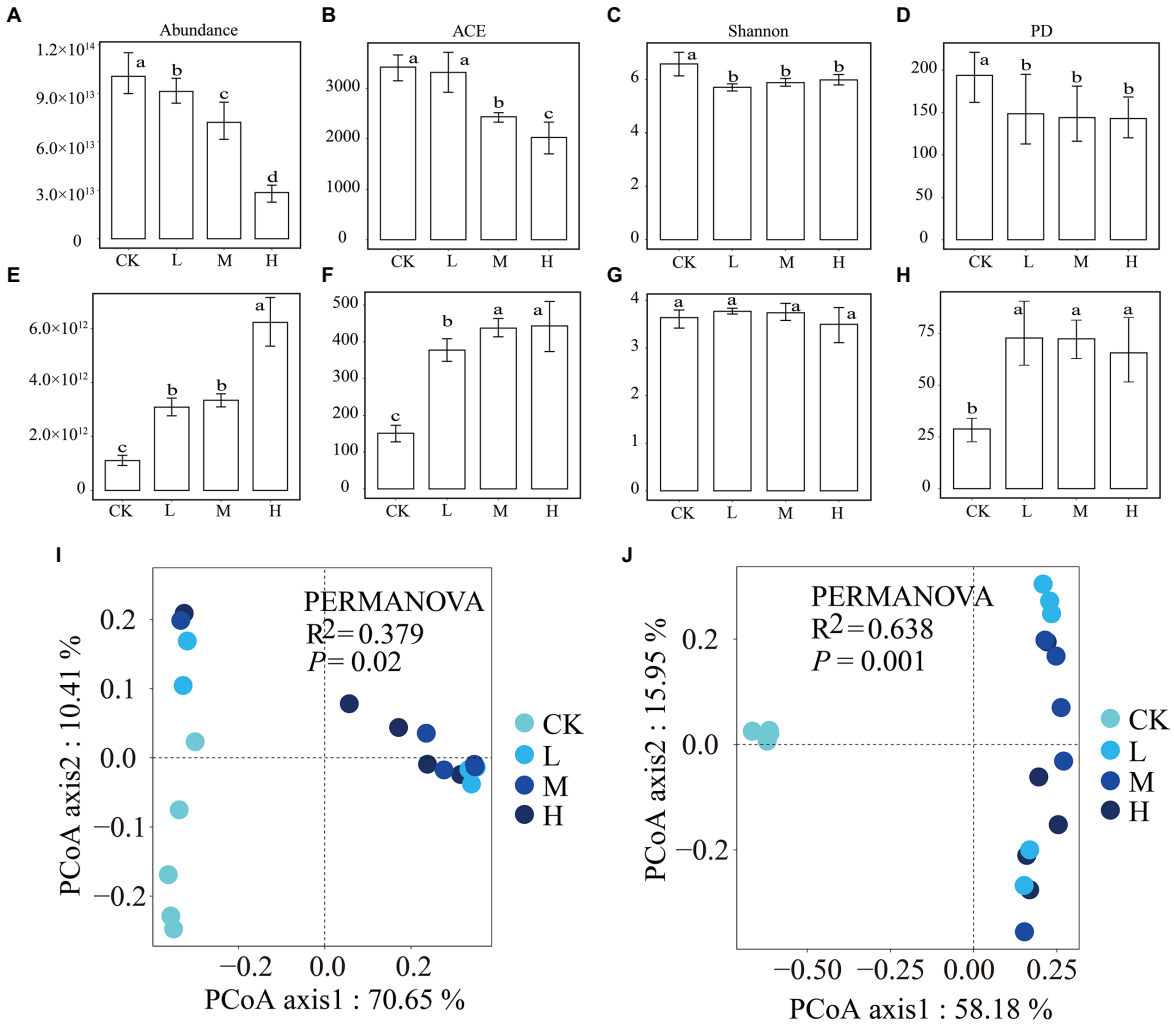
Differences in the abundance, alpha diversity, and beta diversity of bacterial and fungal communities among the different *RI* levels. Abundance of bacteria **(A)** and fungi **(E)**; ACE diversity index of bacteria **(B)** and fungi **(F)**; Shannon diversity index of bacteria **(C)** and fungi **(G)**; phylogenetic diversity index of bacteria **(D)** and fungi **(H)**; and the principal coordinate analysis of bacteria **(I)** and fungi **(J)**. Different lowercase letters indicate significant differences among different *RI* levels (*p* < 0.05).

The qPCR results demonstrated that there was a significant (*p* < 0.05) decreasing trend in bacterial abundance and a significant (*p* < 0.05) increasing trend in fungal abundance with increasing *RI* levels (Figures 1A,E). A total of 767,730 16 s rRNA gene reads and 1,049,494 ITS gene reads were obtained for all the samples, which could be clustered into 4,716 and 983 OTUs, respectively. The alpha diversity results differed for bacteria and fungi. The ACE index for bacteria (Figure 1B) and fungi (Figure 1F) showed trends similar to those of abundance levels. The Shannon (Figure 1C) and PD (Figure 1D) indices for the bacteria were significantly (*p* < 0.05) lower in the HMs treatments than in the CK. The Shannon index for fungi (Figure 1G) showed no change in any of the samples. The PD index (Figure 1H) for HMs treatment was significantly (*p* < 0.05) greater than that for the CK. The PCoA results showed a clear variation in the bacterial (R^2^ = 0.379, *p* = 0.02) and fungal (R^2^ = 0.638, *p* = 0.001) communities between the HMs-treated samples and CK (Figures 1I,J). These variations were also supported by the results of the PERMANOVA tests (*p* < 0.05; Supplementary Table S1).

**Figure 2:**
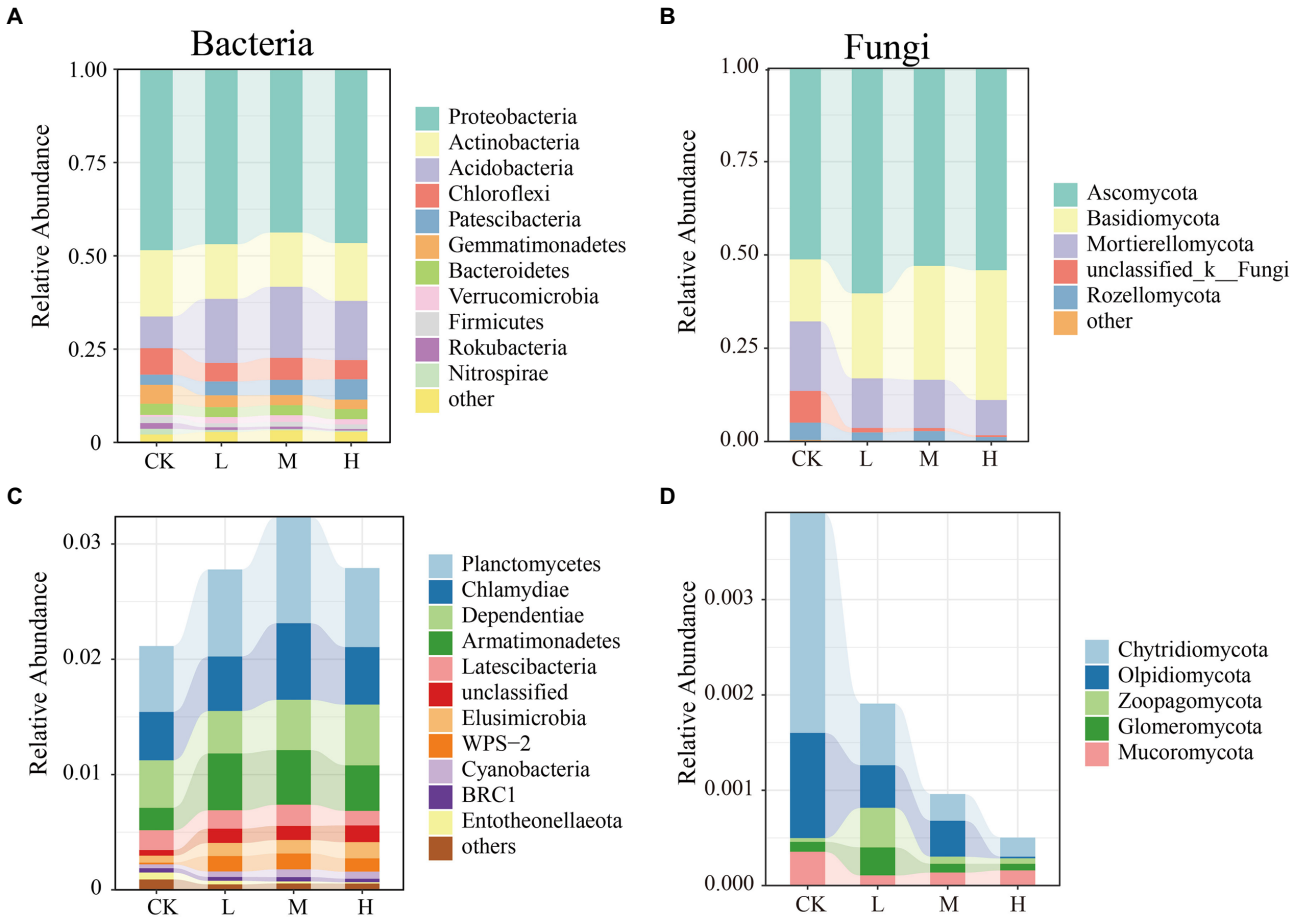
Relative abundance of the bacterial and fungal community phyla at different *RI* levels. Abundant phyla for bacteria **(A)** and fungi **(B)**; Rare phyla for bacteria **(C)** and fungi **(D)**.

The composition of the microbial communities at the phylum level is shown in Figure 2. Across all the samples, the most abundant bacteria phyla were found to be Proteobacteria, Actinobacteria, Acidobacteria, and Chloroflexi, which dominated more than 81% of the total sequences (Figure 2A). HMs treatments enriched the relative abundance of Acidobacteria from 8.49% (CK) to 17.18% (L), 19.04% (M), and 15.86% (H), respectively. The fungal community (Figure 2B) predominantly comprised Ascomycota, Basidiomycota, and Mortierellomycota, which accounted for more than 86% of all fungi. The relative abundance of Basidiomycota was lowest in the CK (16.64%) and increased from 22.74% (L) to 34.75% (H) with increasing *RI* levels. HMs contamination significantly altered the composition of rare microbial phyla. Relative to the rare bacterial phyla (Figure 2C), the relative abundance of Planctomycetes at the L (0.75%), M (0.92%), and H (0.69%) levels was greater than that of the CK (0.57%). For the rare fungal rare phyla, the relative abundances of Chytridiomycota, Olpidiomycota, and Mucoromycota decreased following HMs treatment. At the L level, Zoopagomycota (0.041%) and Glomeromycota (0.029%) had the highest relative abundances (Figure 2D). The heat map results also indicated that HMs stress did not monolithically reduce or enrich the relative abundance of the microbial communities at the genus level (Supplementary Figures S1, S2).

### Networks and stability of microbial communities

**Figure 3:**
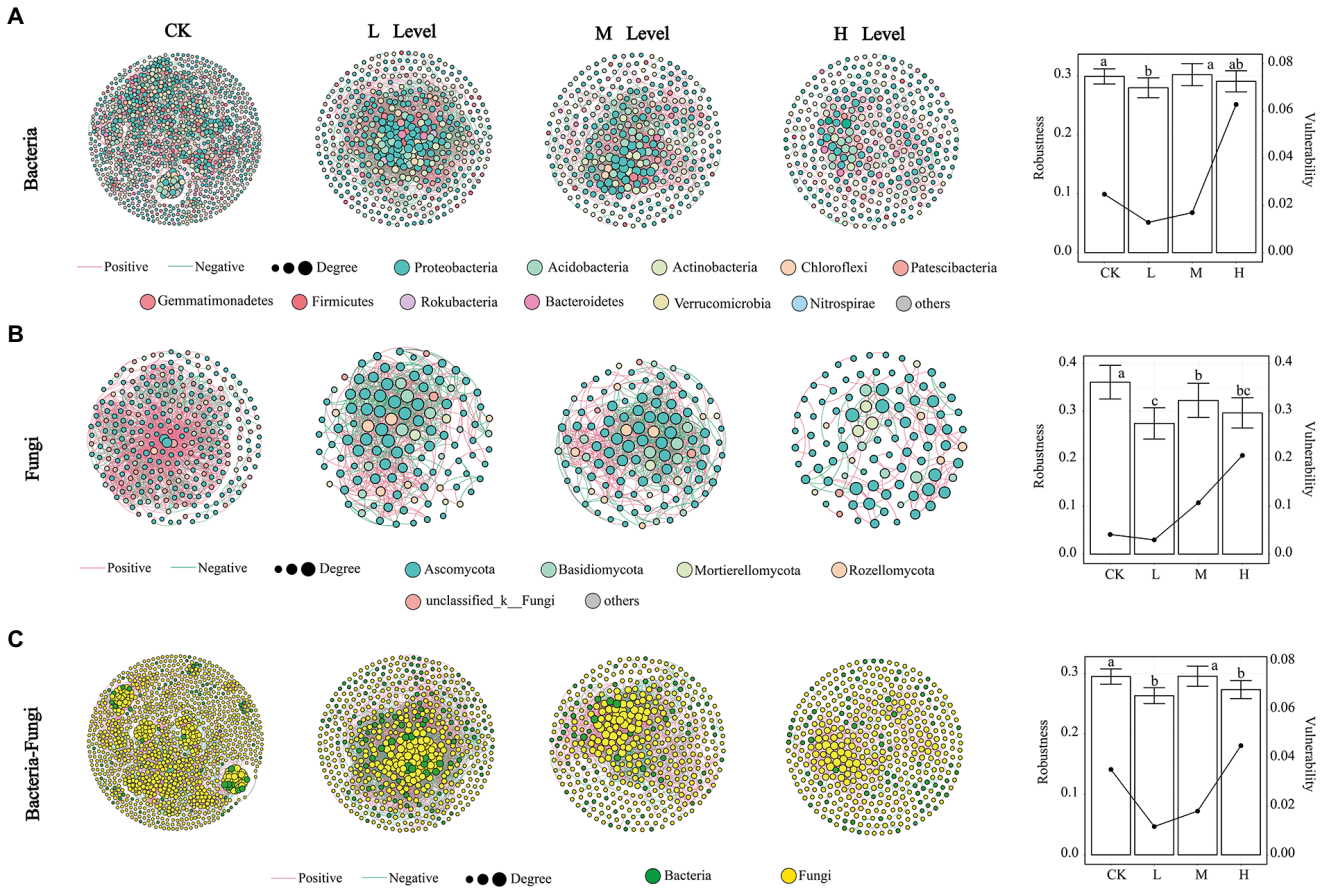
Co-occurrence networks, robustness, and vulnerability of bacterial **(A)**, fungal **(B)**, and bacterial–fungal **(C)** communities. The bar plot represents the robustness. Line graphs represent vulnerability. Different lowercase letters in the bars indicate significant differences among the different *RI* levels (*p* < 0.05).

To investigate the influence of different *RI* levels on microbial community interactions, we generated 12 networks using MENA (Figure 3). The topological properties of the networks are listed in Supplementary Table S2. As the *RI* level increased, simpler networks of microorganisms were observed. Compared with the CK, the nodes, edges, avgCC, GD, and M of the bacterial networks were reduced by the HMs. As the *RI* levels increased, the nodes from 440 to 333, edges from 4,862 to 743, and avgCC from 0.472 to 0.284 of the bacterial networks continued to decrease. Meanwhile, the GD from 3.554 to 6.164 and M from 0.322 to 0.652 showed an upward trend (Supplementary Table S2). The positive correlation ratio of edges also showed an increasing trend, where the M (71.53%) and H (85.89%) levels were greater than those of the CK (66.42%). We calculated the robustness and vulnerability of the network (Figure 3A) to investigate changes in the stability of the bacterial networks under different *RI* levels. The robustness of the M and H levels was greater than that of the L level and not significantly different from the CK. The vulnerability of the H levels was greater than that of the other levels. In the fungal networks, the number of nodes (283) and edges (1,978) was the highest in the CK. The positive correlation ratios for the edges from 56.83% to 77.79%, GD from 3.152 to 4.596, and M from 0.309 to 0.782 showed an increasing trend with increasing *RI* levels (Supplementary Table S3). Robustness was significantly greater in CK than in the other treatments (*p* < 0.05), with a minimum at the L level. Vulnerability was greater at the H level than at the other *RI* levels (Figure 3B).

The symbiotic patterns of soil bacteria and fungi were analyzed under different *RI* levels. Changes in the topological properties, robustness, and vulnerability of the bacterial–fungal networks were similar to those of the bacteria (Figure 3C; Supplementary Table S3). HMs stress led to an increasing trend in the ratio of positive correlations between bacteria and bacteria (B–B) and fungi and fungi (F–F). The proportion of positive correlations between bacteria and fungi (B–F) decreased with increasing *RI* levels, but the ratio was greater than that of CK.

The keystone taxa of the different co-occurrence networks are presented in Supplementary Figure S3. In the bacterial network, 5, 4, 13, and 2 OTUs were defined as keystone taxa, respectively. In the fungal network, there were only 7 OTUs in the CK, which were recognized as keystone taxa. In the bacterial–fungal network, 5, 19, 7, and 4 OTUs were identified as keystone taxa, respectively. The taxonomic information for these keystone taxa is listed in Supplementary Table S3. All the keystone taxa contained rare phyla such as WPS-2, Chytridiomycota, and Armatimonadetes.

### Metabolic characteristics of the microbial communities

Variation in the AWCD for bacterial and fungal communities at different *RI* levels is shown in Supplementary Figure S4. The CK treatment for the bacteria and fungi exhibited higher AWCD than the other treatments throughout the incubation period. The AWCD at 168 h indicated the optimum range of absorbance, which was used to evaluate the functional diversity of the bacteria and fungi.

**Figure 4:**
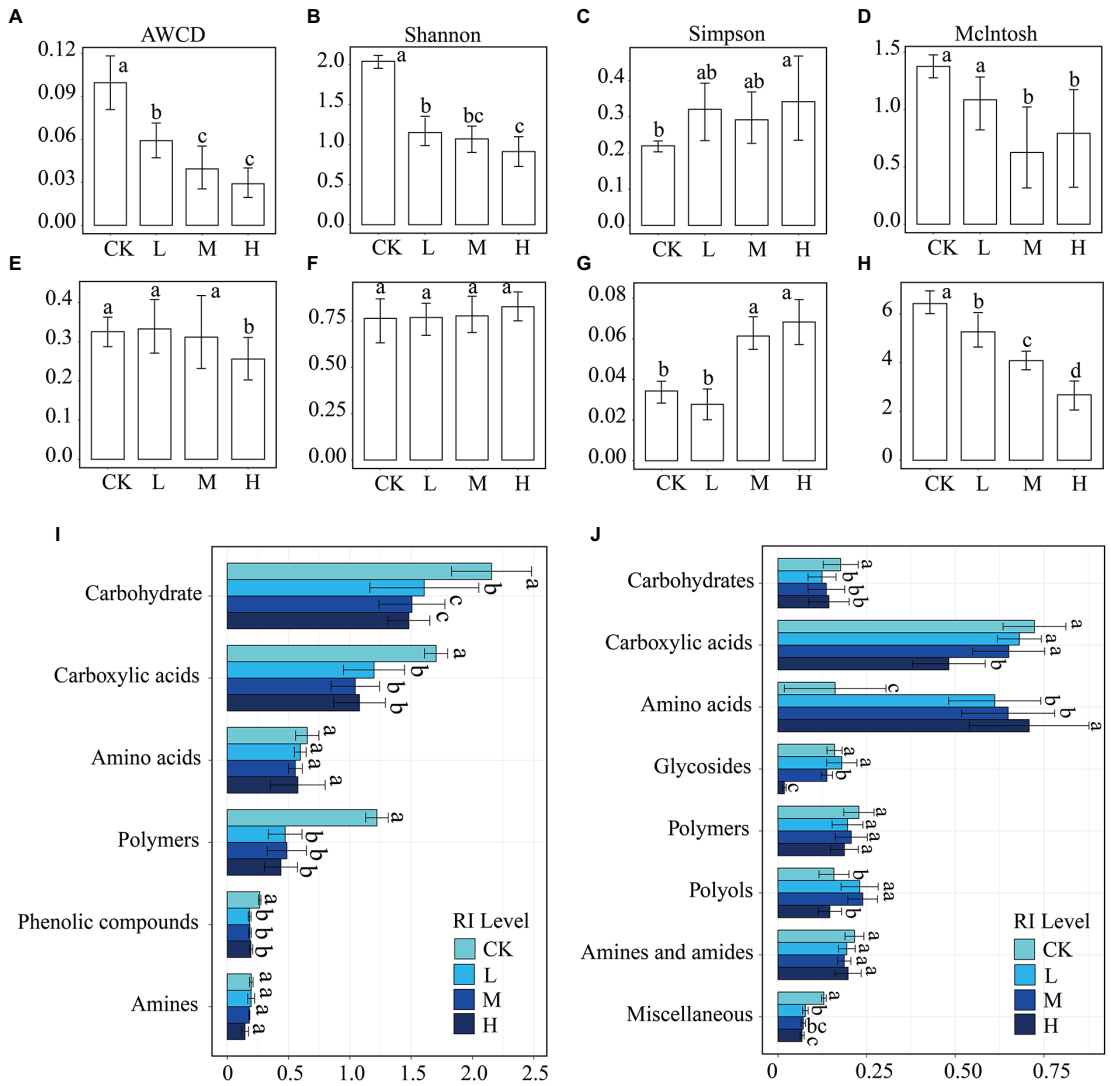
Metabolic profile of bacterial and fungal communities under different *RI* levels. Average well color development (AWCD), Shannon index, Simpson index, and McIntosh index of bacterial **(A–D)** and fungal **(E–H)** communities. Variability among *RI* levels of taxonomic carbon sources for bacteria **(I)** and fungi **(J)**. Different lowercase letters indicate significant differences among different *RI* levels (*p* < 0.05).

For the bacterial communities, increased *RI* levels resulted in significantly reduced AWCD (*p* < 0.05) (Figure 4A), and the lowest value was observed at the H level (0.028). HMs additions significantly (*p* < 0.05) reduced the functional diversity indices (Figures 4B–D) at the H level but did not significantly affect the Simpson and McIntosh indices at the L level (Figures 4C,D). For the fungal communities, there were significant differences (*p* < 0.05) in AWCD between H and the other levels (Figure 4E). The Shannon index (Figure 4F) did not significantly change as the level of *RI* increased, while the McIntosh index (Figure 4H) decreased significantly (*p* < 0.05). The Simpson index for the M and H levels was significantly greater than that for the CK (Figure 4G).

To further investigate how HMs alter the metabolic patterns of soil microorganisms, six bacterial carbon sources (Figure 4I) and eight fungal carbon sources (Figure 4G) were compared at different *RI* levels. HMs addition significantly inhibited (*p* < 0.05) the use intensity of carbohydrates, carboxylic acids, polymers, and phenolic compounds by bacteria when compared to the CK. The use intensity of carbohydrates at the L level was significantly higher than that at the M and H levels (*p* < 0.05). Meanwhile, other carbon sources did not significantly differ among the L, M, and H levels. In the fungal communities, carbohydrates, carboxylic acids, glycosides, polymers, and miscellaneous were used more strongly in the CK than in the L, M, and H levels. However, HMs stress significantly promoted amino acid use (*p* < 0.05). Polymer use also significantly increased at the L and M levels when compared to that at the CK and H levels (*p* < 0.05).

### Responses of microbial functional genes

**Figure 5:**
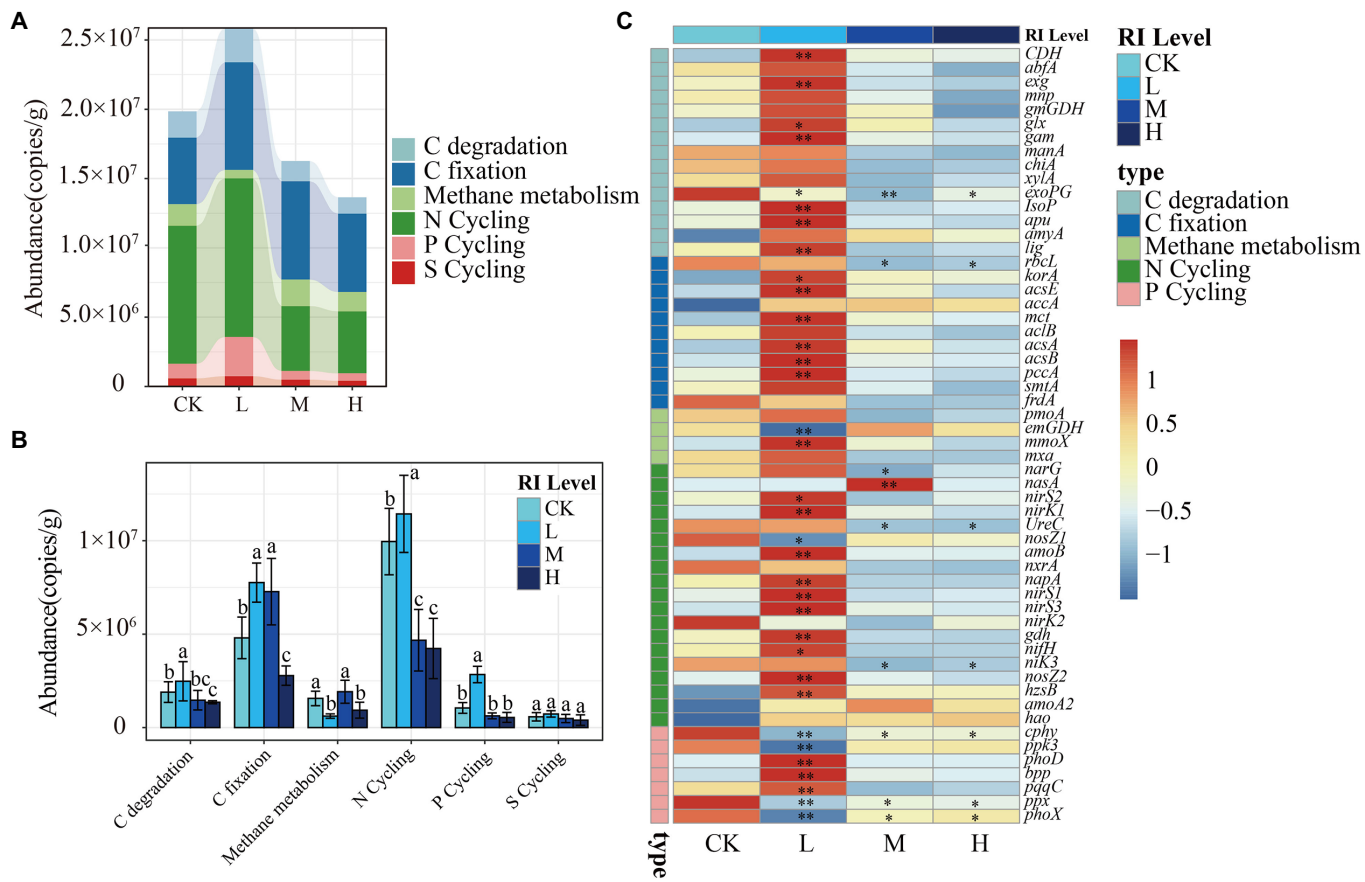
Functional genes. **(A)** The difference in abundance of functional genes under different *RI* levels. **(B)** The abundance of functional genes in the C, N, P, and S cycle. **(C)** The abundance of individual functional genes. Different lowercase letters indicate significant differences among different *RI* levels (*p* < 0.05). The asterisk indicates a significant change in the corresponding genes in this treatment when compared with CK (^*^p < 0.05; ^**^p < 0.01).

To elucidate the responses of microbial functions to HMs, 63 functional genes involved in the carbon cycle (30), nitrogen cycle (20), phosphorus cycle (8), and sulfur cycle (5) were detected across all samples (Figure 5). Functional gene abundance was highest at the L level (2.58 × 10^7^ copies g^−1^), followed by the CK (1.98 × 10^7^ copies g^−1^), M (1.62 × 10^7^ copies g^−1^), and H (1.36 × 10^7^ copies g^−1^) levels (Figure 5A). Compared with that in the CK, HMs stress significantly increased (*p* < 0.05) the gene abundance in relation to C degradation, C fixation, P cycling, and N cycling at the L level. Meanwhile, it showed significant inhibition (*p* < 0.05) at the H level. There were no significant differences in the abundance of S cycle genes (Figure 5B). Further analysis was conducted to investigate the variability of individual functional genes among different *RI* levels (Figure 5C). At the L level, the abundance of 14 genes for C cycling, 10 genes for N cycling, and 3 genes for P cycling, including *cdh* for degrading cellulose, *acs*A and *acs*B involved in the acetyl coenzyme A reduction pathway, and *nir*S1 and *nir*S3 involved in the denitrification functions, were significantly higher (*p* < 0.05). However, the abundance of most genes was reduced at the M and H levels, with 7 genes being significantly repressed (*p* < 0.05). This included the *exoPG* for degrading pectinase, *UreC* for encoding urease, and *ppx* for encoding exopolyphosphatase (Figure 5C).

**Figure 6:**
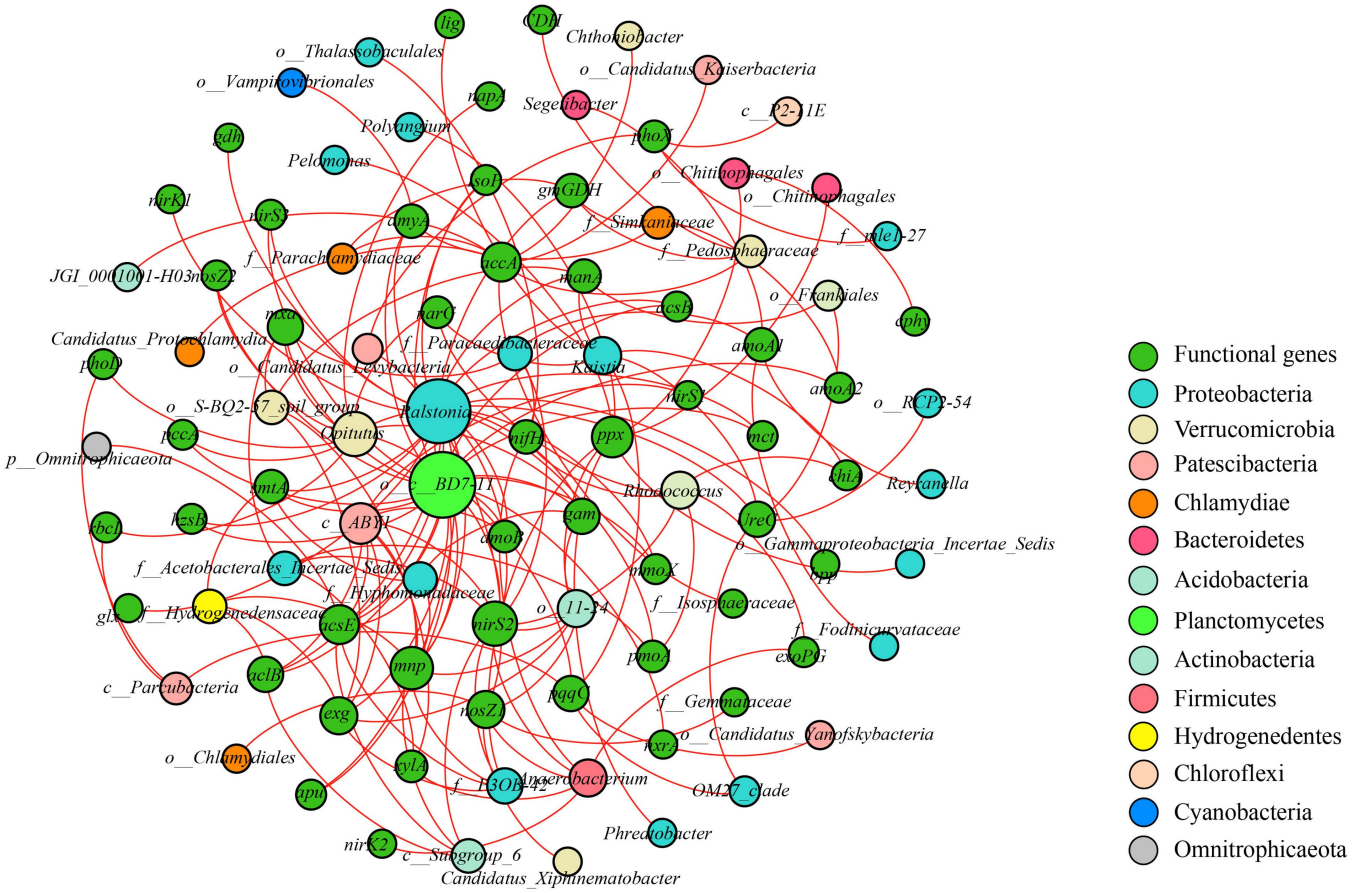
Co-occurrence network of bacterial communities and functional genes. The red links represent positive correlations.

Co-occurrence patterns of the functional genes and bacterial genera were analyzed to explore the links between them. The network only showed positive correlations between microbial communities and functional genes (Figure 6). *o__c__BD7-11*, belonging to Planctomycetes of rare taxa, possessed the highest degree of gene numbers attached to the node. It was positively correlated with 22 genes, including *smtA*, *nirS3*, *acsB*, and *mmoX*. Ralstonia, which belongs to the Proteobacteria of abundant taxa, were also associated with *smtA*, *nirS3*, *acsB*, and *mmoX*. This suggests that abundant and rare taxa may have the same role in coding of functional genes under HMs stress.

### Assembly processes of the microbial community

**Figure 7:**
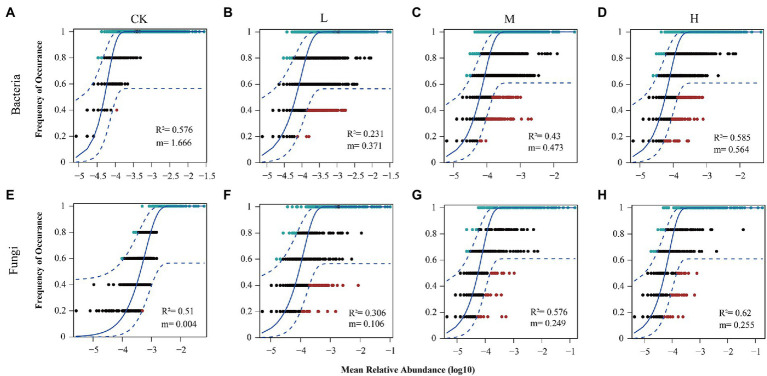
Fit of the NCM for community assembly. Bacterial community assembly at different *RI* levels **(A–D)**. Fungal community assembly at different *RI* levels **(E–H)**. The solid blue line indicates the best fit to Sloan’s neutral model. The dashed blue lines represent 95% confidence intervals around the model prediction. OTUs with frequencies higher and lower than the predicted occurrence frequency by NCM are shown in blue and red, respectively.

**Figure 8:**
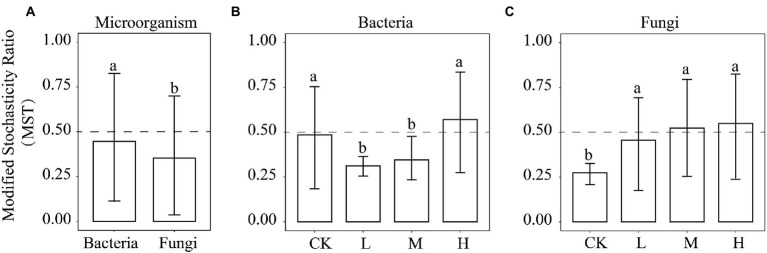
The MST ratio of microorganisms **(A)**, bacteria **(B)**, and fungi **(C)**. The dotted line at 0.5 is the boundary point between more stochastic (MST > 0.5) and deterministic (MST < 0.5) assembly processes. Different lowercase letters indicate significant differences among the different *RI* levels (*p* < 0.05).

To explore the relative importance of deterministic and stochastic processes in shaping the microbial communities at different *RI* levels, bacterial and fungal communities were fitted to the NCM (Figure 7). As the *RI* level increased, the goodness of fit displayed a trend of decreasing and then increasing in the bacterial and fungal communities, with the minimum at the L level (R^2^ = 0.231 and R^2^ = 0.306). The goodness of fit of the bacterial communities was similar between the H level (R^2^ = 0.585) and CK (R^2^ = 0.576). However, the goodness of fit of the fungal communities was greater for the H level (R^2^ = 0.62) than CK (R^2^ = 0.51). The migration rate for the bacterial communities showed the same trend as the goodness of fit, with a maximum for the CK (m = 1.666). However, the migration rate (m = 0.004–0.255) of the fungal communities showed an increasing trend. We further employed MST to determine the roles of deterministic and stochastic processes in the microbial community assembly (Figure 8). HMs additions significantly reduced (*p* < 0.05) the MST values for the bacterial community at the L (0.31) and M (0.34) levels. Meanwhile, no significant differences were found between the CK (0.48) and H (0.57) levels. There was an increasing trend in MST values for the fungal community, and MST values were significantly (*p* < 0.05) higher at the L (0.45), M (0.52), and H (0.54) levels than in the CK (0.27).

## Discussion

*RI* is one of the most used methods for evaluating soil HMs contamination. Our study has shown that the microbial diversity indices, and abundance are more highly correlated with the *RI* levels than the concentrations of Pb and Cd (Li et al., 2022). To date, studies have focused on the responses of soil microorganisms to different concentrations of Pb and Cd. Meanwhile, research on changes in microbial communities at different *RI* levels is lacking. In this study, we conducted soil microcosm analysis at different *RI* levels by Pb and Cd to investigate the responses of their microbial communities.

HMs contamination can alter microbial abundance and community diversity (Qi et al., 2022). The alpha diversity index of the microbial community gradually decreased with the increase concentrations of Pb and Cd (Xiao et al., 2020). Similarly, the abundance and alpha diversity index of bacteria at different *RI* levels were found to be lower than those of the CK (Figure 1). In contrast to bacteria, the abundance and ACE index of fungi were found to increase significantly with *RI* levels (Figure 1). We found that HMs had negative and positive effects on bacterial and fungal PD indices, respectively (Figure 1D). These variations may be attributed to various factors. First, fungi are more resistant to HMs than bacteria because they can produce mycorrhizae and form a link with plant roots to increase resistance (Luo et al., 2014; Qi et al., 2022). Second, the extra carbon released from bacteria killed by the HMs could be used for fungal growth (Rajapaksha et al., 2004). Finally, there may be antagonistic effects between fungi and bacteria, and the reduction in bacteria due to HMs toxicity may eliminate the competitive stress on fungus (Rousk et al., 2008; Fernández-Calviño and Bååth, 2016).

Proteobacteria, Actinobacteria, and Acidobacteria were the dominant phyla for bacteria, and the dominant fungal phyla were Ascomycota and Basidiomycota (Figure 2), which is consistent with the findings of previous studies (Lin et al., 2022; Ma et al., 2022). The relative abundance of Acidobacteria and Basidiomycota increased with *RI* level, which could be related to their adaptations to HMs stress (Figure 2). Acidobacteria is resistant to HMs (Wang et al., 2019) and critical for maintaining balance in ecosystems because they can self-regulate (Chen et al., 2016). Basidiomycota have been used previously to remediate HMs pollution (Hassan et al., 2020). Some of the keystone taxa divided by the Zi–Pi diagram were found to pertain to rare taxa in our study (Supplementary Figure S3; Supplementary Table S7). *o__c__BD7-11*, which belongs to Planctomycetes, possessed the highest degree in the co-occurrence patterns of functional genes and bacterial genera (Figure 6). These results indicate that rare taxa play vital roles in metal(loid) metabolisms and functional diversity (Rocca et al., 2020). Rare taxa can also rapidly respond to maintain community stability when faced with HMs stress (Zuo et al., 2022). Planctomycetes can detoxify HMs by secreting extracellular polymeric substances (Zhang et al., 2021). An increase in the relative abundance of Planctomycetes with rising *RI* levels may reduce the toxic effects of HMs (Figure 2C). The decrease in the relative abundance of a large proportion of fungal rare taxa indicated that HMs may destroy the stability of fungal communities (Figure 2D). This was supported by the increase in vulnerability at different *RI* levels (Figure 3B).

The number of nodes, edges, and avgCC values for the bacterial, fungal, and bacterial–fungal networks reduced with increasing *RI* levels (Figure 3; Supplementary Table S7). High levels of HMs can disrupt cell structures and functions, accelerate cell death, and inhibit microbial activity or competitive ability. This can potentially reduce the complexity of the microbial networks (Shuaib et al., 2021). Compared with CK, the GD and M values increased with increasing *RI* levels. This shows that higher *RI* levels may lead to greater distances and higher modularity among nodes (Wan et al., 2020; Chen et al., 2022). Microorganisms can respond to HMs stress through their interaction (Chun et al., 2021). In the present study, the proportion of positive links in the bacterial, fungal, and bacterial–fungal networks decreased and then increased. This indicates that microorganisms can resist HMs stress through cooperation (Qi et al., 2022). From a microbial community stability perspective, the robustness of the M level was higher than that of the L and H levels. Meanwhile, the vulnerability of the H level was greater than that of the L and M levels (Figure 3). This may be owing to the moderate disturbance effect resulting in a more stable microbial community at the M level (Bao et al., 2022). However, the toxic effects of the HMs at the H level were too high causing the microbial community to rebuild (Chen et al., 2022). Keystone taxa have been proven to significantly contribute to the stability of microbial communities (Banerjee et al., 2018; Liu S. et al., 2022). The smallest numbers of keystone taxa were identified at the H level in all microbial networks, representing further evidence that the H level severely reduces the stability of microbial communities.

Microbial community diversity and composition under HMs stress are closely related to community assembly mechanisms (Sun et al., 2021). Quantifying the relative importance of deterministic and stochastic processes can provide a clearer understanding of microbial community responses to HMs stress (Wang et al., 2021). The MST values of all samples were significantly greater for bacteria than for fungi (*p* < 0.05; Figure 8A). This suggests that stochastic processes contributed more to the bacterial community than to the fungal community. This may be due to the size plasticity hypothesis (body size effect), whereby smaller organisms (bacteria) are less susceptible to environmental filtering than larger organisms (Liu et al., 2015). HMs contamination creates highly selective stress on the microbial community (Laplante et al., 2013). The microbial community assembly tends to be more deterministic with increasing concentrations of HMs (Zhang et al., 2022). However, our results show that the bacterial community assembly at the L and M levels tended to be deterministic, whereas the bacterial community assembly tended to be stochastic at the H level. This may be because HMs stress at H levels exceeds the tolerance range of the bacteria (Hassen et al., 1998). The bacterial diversity and abundance were significantly reduced (Figure 1), and the vulnerability of the bacterial community (Figure 3A) increased, leading to the reconstitution of the bacterial community in which stochastic processes dominate. The ability of microorganisms to diffuse in different environments can also lead to different assembly mechanisms (Walters et al., 2022). Although the addition of Pb and Cd limited the migration rate of the bacteria compared to the CK, the increased migration rate of bacteria with increasing *RI* levels also caused the assembly process to tend toward being stochastic (Figure 7). HMs treatment resulted in fungal community assembly being dominated by stochastic processes, which is in line with previous findings (Liu B. et al., 2022). The toxicity of HMs may affect the growth of fungi, thereby reducing taxon colonization and greater exclusion (Feng et al., 2018). We found no significant differences in MST values among the different *RI* levels (Figure 6). This may be owing to the high tolerance of the fungal community weakening the environmental filtering of different levels of HMs (Wang W. et al., 2022).

Metabolic functions of microorganisms can be regulated to adapt to changes in their surroundings (Tikariha and Purohit, 2019). Xiao et al. (2020) reported that HMs significantly reduce the metabolic activity of bacteria. We also obtained similar findings showing that AWCD was found to decrease significantly with *RI* levels in the bacterial community (Figure 4A). However, the fungal AWCD was significantly reduced only at the H level (Figure 4E). This suggests that the metabolic capacity of fungi is more stable than the bacterial metabolic capacity in HMs contamination (Stefanowicz et al., 2008). This difference may be caused by resistant fungal species that do not need to compete for the same C source as more sensitive bacterial species that are killed or show depleted activity in polluted soils (Møller et al., 1999). The catabolism of carbohydrates, carboxylic acids, and polymers was particularly sensitive to HMs toxicity (Muniz et al., 2014). Bacterial use of these three carbon sources was significantly lower than that in the CK (*p* < 0.05, Figure 4I). For fungi, HMs treatment was found to reduce carbohydrate and carboxylic acid use while increasing amino acid use (Figure 4J). This was likely due to metal stress inducing a variety of cellular detoxification mechanisms in microorganisms, including protein and amino acid macromolecules (Hall, 2002). This can be metabolized by microbial species that are better able to use amino acids than other carbon sources (Kenarova and Boteva, 2015). The effect of HMs on microbial functional diversity is lower with low to moderate soil contamination (Kenarova et al., 2014), and diversity is substantially reduced under high HMs contamination (Gremion et al., 2004). We also found that the functional diversity index of microorganisms at the H level was significantly lower than that in the CK.

The findings of the present study show that the abundance of C, N, and P cycling genes was significantly different among the various *RI* levels, with the smallest total abundance at the H level (Figure 5). However, the level of HMs contamination has no significant effect on functional gene abundance (Luo et al., 2020; Qi Q. et al., 2022). The possible reasons for these disparities are the concentration and type of contaminating metals, such as Pb and Cd, being unnecessary and likely dangerous to microorganisms (Shuaib et al., 2021). Inorganic polyphosphate (polyP) is a highly effective complexing agent for metal ions (Lin et al., 2021). In *Escherichia coli*, cells with high polyP content were more tolerant to Cd^2+^ than those with low polyP levels (Keasling and Hupf, 1996). Meanwhile, enzymes that degrade polyP are also essential for bacterial tolerance to HMs (Keasling et al., 2000). The *ppx* gene encoding exopolyphosphatase to hydrolyze polyP was significantly lower in abundance with HMs treatments than in the CK (Figure 5C). This may account for the decrease in bacterial abundance and diversity (Figure 1). *UreC* is one of the critical genes for bacterial urease production because it encodes the largest alpha subunit of urease (Yin et al., 2021). Urease released by bacteria regulates the hydrolysis of urea to CO_3_^2−^ and NH_4_^+^. NH_4_^+^ releases NH_3_, which increases the pH. Cd^2+^ or Pb^2+^ combines with CO_3_^2−^to form CdCO_3_ or PbCO_3_ precipitate (Han et al., 2020). The reduction in *UreC* abundance at the M and H levels suggests that the urea hydrolysis process is affected, which affects the microbially-induced carbonate precipitation of HMs (Figure 5C).

## Conclusion

In this study, we explored the abundance, structure, diversity, function, metabolism, and assembly processes of microbial communities in response to Pb and Cd stress at different *RI* levels. Bacteria were found to be more sensitive to changes in the *RI* levels than fungi. This was supported by the significant reductions in the bacterial abundance and alpha diversity index with HMs stress, and the considerable decrease in AWCD of the bacteria as the *RI* levels increased. However, the abundance and ACE index of the fungi increased significantly with *RI* level, and the fungal AWCD was only substantially reduced with the H level. The rise in *RI* levels disturbed the relative abundance of the microbially abundant and rare taxa, including Acidobacteria, Basidiomycota, and Planctomycetes. Analysis of the keystone taxa and co-occurrence patterns showed that rare taxa play a vital role in microbial community stability and function. The number of key taxa was lowest at the H level, and we found that the complexity and vulnerability of the microbial network decreased as the *RI* level increased. Meanwhile, the intraspecific cooperation of the microbial community was enhanced to cope with HMs stress. Metabolically, HMs treatment reduced carbohydrate and carboxylic acid use by bacteria and fungi, and the functional diversity index of the microorganisms at the H level was significantly lower. Functional analysis suggested that the abundance of the C, N, and P cycling genes was significantly different among the various *RI* levels, with the lowest abundance being found at the H level. We also found that the microbial community assembly tended to be more stochastic as the *RI* level increased. However, this requires further investigation. This study has expanded on our previous research and provided a detailed examination of microbial responses to Pb and Cd contamination.

## Data availability statement

The data presented in the study are deposited in the NCBI repository, accession number PRJNA899377.

## Author contributions

DL conducted the experiment and wrote the main manuscript. JC helped with the experiment design and prepared data analytical methods. XZ helped with the soil microcosm experiment. WS helped with the writing and revision of the language. JL was responsible for project administration and funding acquisition. All authors contributed to the article and approved the submitted version.

## Funding

This study was supported by the National Natural Science Foundation of China (grant nos. U1910207 and 41771548) and the Natural Science Basic Research Program of Shanxi Province (grant no. 202103021224031).

## Conflict of interest

The authors declare that the research was conducted in the absence of any commercial or financial relationships that could be construed as a potential conflict of interest.

## Publisher’s note

All claims expressed in this article are solely those of the authors and do not necessarily represent those of their affiliated organizations, or those of the publisher, the editors and the reviewers. Any product that may be evaluated in this article, or claim that may be made by its manufacturer, is not guaranteed or endorsed by the publisher.

## Footnotes

^1^ https://cloud.majorbio.com

^2^ http://ieg4.rccc.ou.edu/MENA
