# Supplementary material for: Structural and functional characteristics of soil microbial communities in response to different ecological risk levels of heavy metals
Source: Front Microbiol. 2022 Dec 8;13:1072389. doi: 10.3389/fmicb.2022.1072389 (PMC9772559; doi:10.3389/fmicb.2022.1072389)
Supplement: Supplementary file 1 [file Data_Sheet_1.docx]

**Table S1** **Basic properties of the soil used for the soil microcosm setup**

| Soil physicochemical characteristics | Values |
| --- | --- |
| As (mg kg^-1^) | 26.01 ± 3.05 |
| Cd (mg kg^-1^) | 0.07 ± 0.02 |
| Cr (mg kg^-1^) | 102.89 ± 3.63 |
| Cu (mg kg^-1^) | 42.16 ± 4.93 |
| Ni (mg kg^-1^) | 36.89 ± 1.74 |
| Pb (mg kg^-1^) | 28.91 ± 0.78 |
| Zn (mg kg^-1^) | 97.84 ± 15.08 |
| Total nitrogen (TN) (g kg^-1^) | 79.63 ± 22.43 |
| Total carbon (TC) (g kg^-1^) | 14.27 ± 0.05 |
| Total sulfur (g kg^-1^) | 0.31 ± 0.08 |

Data are means ± standard deviation

**Table S2** **Classification of the potential ecological risks based on the *RI* thresholds**

| *RI* thresholds | Degree of risk |
| --- | --- |
| *RI* < 150 | Low risk |
| 150 ≤ *RI* < 300 | Moderate risk |
| 300 ≤ *RI* <600 | Considerate risk |
| 600 ≤ *RI* | Very high risk |

**Table S3 Formulas for calculations of the RI and microbial functional diversity index**

| Index | Formulae | Definitions |
| --- | --- | --- |
| Potential ecological risk index (*RI*) | $RI=\sum_{i}^{n} E_{r}^{i}=\sum_{i}^{n} \left( T_{r}^{i}\times\frac{C_{D}^{i}}{C_{R}^{i}} \right)$ | $C_{D}^{i}$ and $C_{R}^{i}$ are the measured concentrations of heavy metal *i* in the sample and its background reference value (mg⋅kg^−1^), respectively. $T_{r}^{i}$ represents the TF for heavy metal *i*. $E_{r}^{i}$ denotes the single potential ecological risk factor for heavy metal *i*. |
| Average well color development (AWCD) | $\mathrm{AWCD}=\frac{\sum_{i=1}^{N} {OD}_{i}}{N}$ | OD = C-R, where C is the absorbance value in the control cells and R the is absorbance value in each carbon source well. N is the number of substrates, which in this case is N = 31 (for Biolog ECO plate) or N = 95(for Biolog FF plate). $P_{i}$ is the ratio of the average absorbance value of the i-th well to the sum of that of all the wells in the plate. |
| Shannon-Wiener diversity index (*H’*) | $H^{'}=-\sum_{i=1}^{N} (P_{i}{InP}_{i})$ |  |
| Simpson diversity index (*D*) | $D=1-\sum_{i=1}^{N} P_{i}^{2}$ |  |
| Mclntosh diversity index (*U*) | $U=\sqrt{\sum_{i=1}^{N} {({OD}_{i})}^{2}}$ |  |

**Table S4** **Treatments for the soil microcosms**

| *RI* levels | Simple | $E_{r}^{i}$ | The content of Pb/Cd/ (mg/kg) |
| --- | --- | --- | --- |
| CK | CK | Pb (5)/Cd (30) | Pb (28.81)/Cd (0.07) |
| L: *RI* = 100 | L1 | Pb (5)/Cd (95) | Pb (28.81)/Cd (0.21) |
|  | L2 | Pb (20)/Cd (80) | Pb (115.24)/Cd (0.19) |
|  | L3 | Pb (40)/Cd (60) | Pb (230.48)/Cd (0.14) |
|  | L4 | Pb (60)/Cd (40) | Pb (345.72)/Cd (0.09)/ |
|  | L5 | Pb (70)/Cd (30) | Pb (403.34)/Cd (0.07) |
| M: *RI* = 200 | M1 | Pb (5)/Cd (195) | Pb (28.81)/Cd (0.44) |
|  | M2 | Pb (40)/Cd (160) | Pb (230.48)/Cd (0.37) |
|  | M3 | Pb (80)/Cd (120) | Pb (460.96)/Cd (0.28) |
|  | M4 | Pb (120)/Cd (80) | Pb (691.44)/Cd (0.19) |
|  | M5 | Pb (160)/Cd (40) | Pb (921.92)/Cd (0.09) |
|  | M6 | Pb (170)/Cd (30) | Pb (979.54)/Cd (0.07) |
| H: *RI* = 400 | H1 | Pb (5)/Cd (395) | Pb (28.81)/Cd (0.88) |
|  | H2 | Pb (80)/Cd (320) | Pb (460.96)/Cd (0.75) |
|  | H3 | Pb (160)/Cd (240) | Pb (921.92)/Cd (0.56) |
|  | H4 | Pb (240)/Cd (160) | Pb (1382.88)/Cd (0.37) |
|  | H5 | Pb (320)/Cd (80) | Pb (1843.84)/Cd (0.19) |
|  | H6 | Pb (370)/Cd (30) | Pb (2131.94)/Cd (0.07) |

**Table S5 Target genes and primer sequences used in this study**

| Classification | Gene | Forward sequence (5’ → 3’) | Reverse sequence (5’ → 3’) |
| --- | --- | --- | --- |
| Bacteria | 16S rRNA | 338F (ACTCCTACGGGAGGCAGCAG) | 806R (GGACTACHVGGGTWTCTAAT) |
| Fungi | ITS | ITS1F (CTTGGTCATTTAGAGGAAGTAA) | ITS2R (GCTGCGTTCTTCATCGATGC) |
| C degradation | *abfA* | CGSTAYCCSGGCGGCAAYTT | TGCCASGGNCCGTCCATYTC |
|  | *amyA* | YGGTTTTCGTCTTGACGCSG | MGGCTGMGTRTCATGRTTK |
|  | *amyX* | TATAAYTGGGGMTATGAYCC | CCCATYAAATCAAAWCGRAA |
|  | *apu* | ACVTGGATAGGYGAGCCYCA | CCRTCSGGGAAGTAGTTKCC |
|  | *CDH* | ATWRYCTWCCGMRTHGCCMT | GTKAGSGGRTTBYKGRYCAT |
|  | *exg* | YSTACGGSATGCACTGGMT | TANCGCAGRTAGTCVCCCAT |
|  | *chiA* | TSAAGAARTACGCSGACAACG | ASGTCATCAGRCCCTTSAG |
|  | *exc* | GATTGGTSVCAATATGAYRG | STCCARCCACCRAYRCTRAA |
|  | *glx* | AACCAGTCGATCATCTACGA | RTGSACGAGCTCDGGCATGG |
|  | *IsoP* | GTCATYTACTTYGGNCC | CGNGCSACATCNGCCCA |
|  | *lig* | CCGCACACACTGTTGCTGC | CGAAGGATTGCCACTCGCA |
|  | *manA* | ATGCGCGGBGTCAACCA | TCGTTGSCGATGTTGABGA |
|  | *mnp* | MACRCCSTTCGACTCSACC | ACGTCSGAGCAGTCRAYGA |
|  | *naglu* | TVAAYTGGTAYCTGAAATAY | CCRTGYAGVGCCATCCAGTC |
|  | *exoPG* | ANCATTGGTGGCCSTGGAA | TTRAYGGCRATRCARTCRTC |
|  | *pox* | ACYAGTATCCATTGGCACGGT | AGATGVGARTGATACCARAA |
|  | *gam* | CGSAACTGGGAYTACCGS | TCCCACAGSCCSKCGTC |
|  | *xylA* | TGGGGBGGTCGYGAAGG | ACTTTGGCRTCRAAGTT |
| C fixation | *accA* | GAAGGCTAYCGCAA RGC | CCTTCMGGSGARATMAC |
|  | *aclB* | TGGACMATGGTDGCYGGKGGT | ATAGTTKGGSCCACCTCTTC |
|  | *acsA* | GATACCTGGTGGCAGACCGA | TGATCACGTCGTCGACCCGG |
|  | *acsB* | CTYTGYCAGTCMTTYGCBCC | CCCATAAABCCYGGDGTYTG |
|  | *acsE* | TCATCGGCGAACGCATCAAC | AGRCCGGCTTCSATGGC |
|  | *cdaR* | CGARATGGTGGTGCTCAA | CARCGTRTTACGATGAATA |
|  | *frdA* | MTGCTGCACACSCTGTW | CCGGTSGGGTGRWACTG |
|  | *korA* | GCCGGCTACCCCATCACCCC | ATGATGGGATGGTCGCCATG |
|  | *mct* | TGGGCGCSGASGTSATMCG | TTGACSGTRTARTCSAYSGC |
|  | *mcrA* | GGTGGTGTMGGDTTCACMCARTA | CGTTCATBGCGTAGTTVGGRTAGT |
|  | *pccA* | GTGMTGATCAAGGCCWC | CGSGTGTTCATYTCSAGGAA |
|  | *rbcL* | AAGGAYGACGAGAACATC | TGCAGSATCATGTCRTT |
|  | *smtA* | TTTCTGGCCGGBTAYGCDGC | CGGTACGGHCCGGTYTGVCC |
| Methane metabolism | *mmoX* | ATGGAGGCGGTCAAGGACGA | CGCTTCATGCCCTTCCACAG |
|  | *mxa* | GCGGCACCAACTGGGGCTGGT | GGGCAGCATGAAGGGCTCCC |
|  | *emGDH* | TGTTCTATGTGCCGG CCAA | CTTCCACAGTTCCTTG CC |
|  | *pmoA* | GGNGACTGGGACTTCTGG | GAASGCNGAGAAGAASGC |
| N Cycling | *amoA1* | STAATGGTCTGGCTTAGACG | GCGGCCATCCATCTGTATGT |
|  | *amoA2* | GGGGTTTCTACTGGTGGT | CCCCTCKGSAAAGCCTTCTT |
|  | *amoB* | TGGTAYGACATKAWATGG | RCGSGGCARGAACATSGG |
|  | *gdh* | GCCATCGGYCCWTACAAGGG | ATGTCRCCNGCCGGAACGTC |
|  | *hao* | TGTCACATGGGTGTAGACCA | ACCTGGAACATACCCAT |
|  | *hzo* | AAGACNTGYCAYTGGGGWAAA | GACATACCCATACTKGTRTANACNGT |
|  | *hzsA* | WTYGGKTATCARTATGTAG | AAABGGYGAATCATARTGGC |
|  | *hzsB* | ARGGHTGGGGHAGYTGGAAG | GTYCCHACRTCATGVGTCTG |
|  | *napA* | CTGGACIATGGGYTTIAACCA | CCTTCYTTYTCIACCCACAT |
|  | *narG* | TAYGTSGGGCAGGARAAACTG | CGTAGAAGAAGCTGGTGCTGT |
|  | *nasA* | CARCCNAAYGCNATGGG | ATNGTRTGCCAYTGRTC |
|  | *nifH* | AAAGGYGGWATCGGYAARTCCACCAC | TGSGCYTTGTCYTCRCGGATBGGCAT |
|  | *nirK1* | GGMATGGTKCCSTGGCA | GCCTCGATCAGRTTRTGGTT |
|  | *nirK2* | ATGGCGCCATCATGGTNYTNCC | TCGAAGGCCTCGATNARRTTRTG |
|  | *niK3* | TGCACATCGCCAACGGNATGTWYGG | GGCGCGGAAGATGSHRTGRTCNAC |
|  | *nirS1* | GTSAACGTSAAGGARACSGG | GASTTCGGRTGSGTCTTGA |
|  | *nirS2* | ATCGTCAACGTCAARGARACVGG | TTCGGGTGCGTCTTSABGAASAG |
|  | *nirS3* | TGGAGAACGCCGGNCARGTNTGG | GATGATGTCCACGGCNACRTANGG |
|  | *nosZ1* | CGCRACGGCAASAAGGTSMSSGT | CAKRTGCAKSGCRTGGCAGAA |
|  | *nosZ2* | CGYTGTTCMTCGACAGCCAG | CGSACCTTSTTGCCSTYGCG |
|  | *nxrA* | CAGACCGACGTGTGCGAAAG | TCCACAAGGAACGGAAGGTC |
|  | *UreC* | AAGMTSCACGAGGACTGGGG | AGRTGGTGGCASACCATSAGCAT |
| P Cycling | *bpp* | GACGCAGCCGAYGAYCCNGCNI TNTGG | CAGGSCGCANRTCIACRTTRTT |
|  | *cphy* | GTGGACCTRCGRMA RGARWCICA | GTCCGACCATTGCCTGCYTCR CARTGRAMRTGIADCCA |
|  | *gmGDH* | ATCGCGTTCGGGCCGGACG | ATSAGRTTSAGCTCGTCCCA |
|  | *phnK* | CATCGTCGGCGAATCCGG | TGCTGCATGCCGCCGGAAAA |
|  | *phoD* | CAGTGGGACGACCACGAGGT | GAGGCCGATCGGCATGTCG |
|  | *phoX* | GARGAGAACWTCCACGGYTA | GATCTCGATGATRTGRCCRAAG |
|  | *ppk3* | GACCCGAABGTRCTBGCSAT | TTATAATTNCCSGTNCCNA |
|  | *ppx* | TGCATCTGGCGGACGGCCT | AGATCCGCCGCCAATATCA |
|  | *pqqC* | AACCGCTTCTACTACCAG | GCGAACAGCTCGGTCAG |
| S Cycling | *apsA* | GGGYCTKTCCGCYATCAAYAC | ATCATGATCTGCCAGCGGCCGGA |
|  | *dsrA* | ACSCACTGGAAGCACG | GGTGGAGCCGTGCATGTT |
|  | *dsrB* | CAACATCGTYCAYACCCAGGG | GTGTAGCAGTTACCGCA |
|  | *SoxY* | ATCGATGACAACCCCGTGCC | AGCTGGTCCATCTGCATGCCG |
|  | *YedZ* | CTGCTGATCACGCTGGCCAT | GCGATGCAGCTTCTTCCAGCG |

**Table S6.** **Dissimilarity test by PERMANOVA based on Bray-Curtis dissimilarity among different *RI* levels. **P* < 0.05, ***P* < 0.01.**

|  | RI level | R^2^ | *P* |
| --- | --- | --- | --- |
| Bacteria | CK/L | 0.403 | 0.013* |
|  | CK/M | 0.558 | 0.008** |
|  | CK/H | 0.499 | 0.009** |
|  | L/M | 0.047 | 0.819 |
|  | L/H | 0.057 | 0.685 |
|  | M/H | 0.063 | 0.505 |
| Fungi | CK/L | 0.710 | 0.013* |
|  | CK/M | 0.737 | 0.011* |
|  | CK/H | 0.685 | 0.012* |
|  | L/M | 0.071 | 0.557 |
|  | L/H | 0.201 | 0.176 |
|  | M/H | 0.097 | 0.494 |

**Table S7. Topological properties of the microbial co-occurrence networks in different *RI* levels.**

|  | *RI* level | Nodes | Links | Average clustering coefficient (avgCC) | Average path distance (GD) | Modularity (M) | Proportion of positive correlations (link) |
| --- | --- | --- | --- | --- | --- | --- | --- |
| Bacteria | CK | 1078 | 6863 | 0.593 | 7.877 | 0.777 | 66.42% |
|  | L | 440 | 4862 | 0.472 | 3.554 | 0.322 | 57.14% |
|  | M | 336 | 1900 | 0.421 | 4.294 | 0.452 | 71.53% |
|  | H | 333 | 743 | 0.284 | 6.164 | 0.652 | 85.89% |
| Fungi | CK | 283 | 1978 | 0.530 | 2.086 | 0.438 | 73.55% |
|  | L | 114 | 753 | 0.575 | 3.152 | 0.309 | 56.83% |
|  | M | 89 | 293 | 0.393 | 4.020 | 0.454 | 79.86% |
|  | H | 97 | 163 | 0.411 | 4.596 | 0.782 | 77.79% |
| Bacteria-Fungi | CK | 1167 | 7817 | 0.576 | 8.533 | 0.756 | 64.18% (B-B 58.32%;  F-F 0.48%;  B-F 5.37%) |
|  | L | 566 | 6837 | 0.459 | 4.094 | 0.339 | 55.71% (B-B 40.58%;  F-F 1.46%;  B-F 13.66%) |
|  | M | 415 | 2414 | 0.406 | 4.365 | 0.447 | 71.21% (B-B 56.29%;  F-F 2.19%;  B-F 12.71%) |
|  | H | 426 | 897 | 0.298 | 6.420 | 0.707 | 82.18% (B-B 71.12%;  F-F 2.01%;  B-F 9.14%) |

B-B, F-F, and B-F indicate the positive correlations between bacteria and bacteria, fungi and fungi, and bacteria and fungi, respectively.

**Table S8 Lists of keystone taxa in the co-occurrence network of microorganisms**

|  | *RI* level | OTU ID | Zi | Pi | Types | Taxonomy |
| --- | --- | --- | --- | --- | --- | --- |
| Bacteria | CK | OTU4022 | -1.165 | 0.666 | connectors | Proteobacteria |
|  | CK | OTU4615 | -1.114 | 0.64 | connectors | Proteobacteria |
|  | CK | OTU2674 | -1.165 | 0.64 | connectors | Patescibacteria |
|  | CK | OTU1460 | -1.28 | 0.625 | connectors | Proteobacteria |
|  | CK | OTU2617 | -1.63 | 0.625 | connectors | Proteobacteria |
|  | L | OTU3508 | 0.224 | 0.642 | connectors | Bacteroidetes |
|  | L | OTU2484 | -0.455 | 0.636 | connectors | Proteobacteria |
|  | L | OTU1395 | 0.026 | 0.627 | connectors | Armatimonadetes |
|  | L | OTU1088 | -0.37 | 0.624 | connectors | Proteobacteria |
|  | M | OTU2452 | 1.101 | 0.661 | connectors | Acidobacteria |
|  | M | OTU4372 | 0.16 | 0.655 | connectors | Bacteroidetes |
|  | M | OTU2805 | 0.042 | 0.655 | connectors | Actinobacteria |
|  | M | OTU3733 | 0.042 | 0.653 | connectors | Acidobacteria |
|  | M | OTU1636 | 1.257 | 0.646 | connectors | Proteobacteria |
|  | M | OTU2254 | 0.245 | 0.646 | connectors | Acidobacteria |
|  | M | OTU2782 | 1.055 | 0.633 | connectors | Acidobacteria |
|  | M | OTU2256 | -0.906 | 0.633 | connectors | WPS-2 |
|  | M | OTU4615 | 0.245 | 0.631 | connectors | Proteobacteria |
|  | M | OTU2404 | -0.524 | 0.625 | connectors | Proteobacteria |
|  | M | OTU3098 | -0.75 | 0.625 | connectors | Chloroflexi |
|  | M | OTU2897 | -1.533 | 0.625 | connectors | Patescibacteria |
|  | M | OTU4102 | -0.592 | 0.622 | connectors | Proteobacteria |
|  | H | OTU1627 | 0 | 0.64 | connectors | Proteobacteria |
|  | H | OTU2406 | 2.752 | 0.429 | module hubs | Proteobacteria |
| Fungi | CK | OTU777 | 6.851224 | 0.62925 | network hubs | Ascomycota |
|  | CK | OTU458 | 4.610933 | 0.651473 | network hubs | Ascomycota |
|  | CK | OTU648 | -1.09739 | 0.657778 | connectors | Chytridiomycota |
|  | CK | OTU324 | 2.116838 | 0.644557 | connectors | Rozellomycota |
|  | CK | OTU770 | -1.11082 | 0.64 | connectors | unclassified_k__Fungi |
|  | CK | OTU749 | -1.25753 | 0.639053 | connectors | unclassified_k__Fungi |
|  | CK | OTU765 | -1.06719 | 0.628099 | connectors | unclassified_k__Fungi |
| Bacteria-Fungi | CK | B-OTU4022 | -1.147 | 0.666 | connectors | Proteobacteria |
|  | CK | B-OTU492 | -1.869 | 0.656 | connectors | Proteobacteria |
|  | CK | B-OTU1785 | -1.824 | 0.625 | connectors | Bacteroidetes |
|  | CK | B-OTU1460 | -1.28 | 0.625 | connectors | Proteobacteria |
|  | CK | B-OTU3963 | -1.281 | 0.625 | connectors | Actinobacteria |
|  | L | B-OTU1450 | 1.259 | 0.662 | connectors | Actinobacteria |
|  | L | B-OTU2526 | -0.815 | 0.661 | connectors | Proteobacteria |
|  | L | BOTU1486 | 0.175 | 0.661 | connectors | Proteobacteria |
|  | L | F-OTU871 | 1.108 | 0.658 | connectors | Rozellomycota |
|  | L | B-OTU3640 | 0.918 | 0.655 | connectors | Patescibacteria |
|  | L | F-OTU529 | 0.382 | 0.649 | connectors | Ascomycota |
|  | L | B-OTU2245 | -0.325 | 0.647 | connectors | Proteobacteria |
|  | L | B-OTU1395 | 0.205 | 0.642 | connectors | Armatimonadetes |
|  | L | B-OTU2961 | -0.325 | 0.638 | connectors | Proteobacteria |
|  | L | B-OTU4505 | -0.59 | 0.634 | connectors | Acidobacteria |
|  | L | B-OTU4721 | -0.413 | 0.631 | connectors | Patescibacteria |
|  | L | B-OTU3603 | 0.957 | 0.631 | connectors | Proteobacteria |
|  | L | B-OTU1635 | 1.108 | 0.63 | connectors | Proteobacteria |
|  | L | B-OTU2808 | -0.767 | 0.625 | connectors | Proteobacteria |
|  | L | F-OTU876 | -0.392 | 0.625 | connectors | Ascomycota |
|  | L | F-OTU192 | 1.108 | 0.624 | connectors | Ascomycota |
|  | L | F-OTU893 | -0.815 | 0.623 | connectors | Ascomycota |
|  | L | B-OTU4432 | -0.249 | 0.622 | connectors | Proteobacteria |
|  | L | B-OTU2984 | 2.506 | 0.256 | module hubs | Proteobacteria |
|  | M | B-OTU3656 | 1.121 | 0.656 | connectors | Actinobacteria |
|  | M | BOTU1635 | 0.281 | 0.653 | connectors | Proteobacteria |
|  | M | B-OTU1415 | 0.267 | 0.641 | connectors | Proteobacteria |
|  | M | B-OTU2942 | -1.023 | 0.64 | connectors | Proteobacteria |
|  | M | B-OTU2897 | -1.56 | 0.625 | connectors | Patescibacteria |
|  | M | B-OTU457 | -0.817 | 0.625 | connectors | Actinobacteria |
|  | M | B-OTU2135 | 2.576 | 0.623 | network hubs | Actinobacteria |
|  | H | B-OTU4117 | 0.037 | 0.653 | connectors | Acidobacteria |
|  | H | F-OTU33 | -0.916 | 0.64 | connectors | Ascomycota |
|  | H | B-OTU2406 | 2.784 | 0.415 | module hubs | Proteobacteria |
|  | H | B-OTU382 | 2.505 | 0.152 | module hubs | Acidobacteria |

Module hubs are identified as Zi > 2.5, Pi ≤ 0.62, connectors are identified as Zi ≤ 2.5, Pi > 0.62, network hubs are identified as Zi > 2.5, Pi > 0.62.


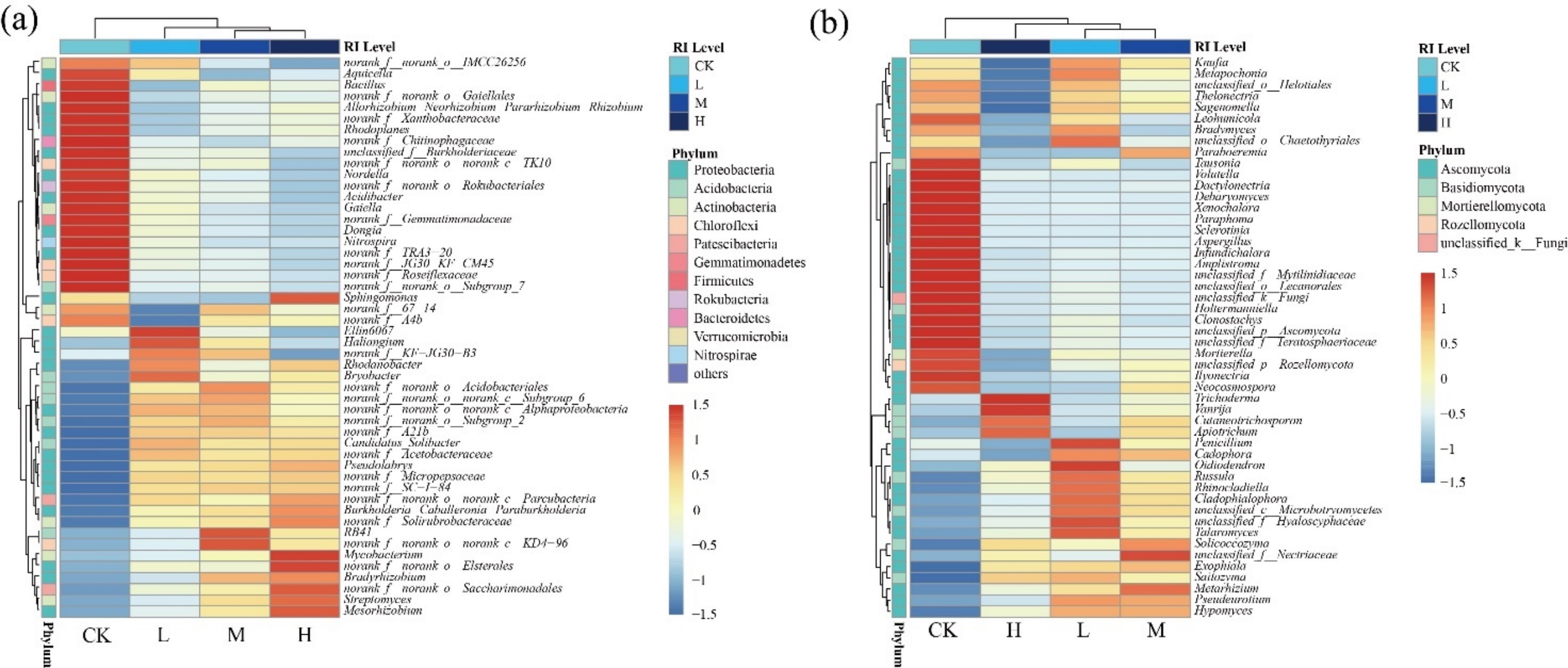


**Fig. S1.** **Heat map showing the relative abundance of the top 50 genera of the abundant bacterial (a) and fungal (b) taxa.**


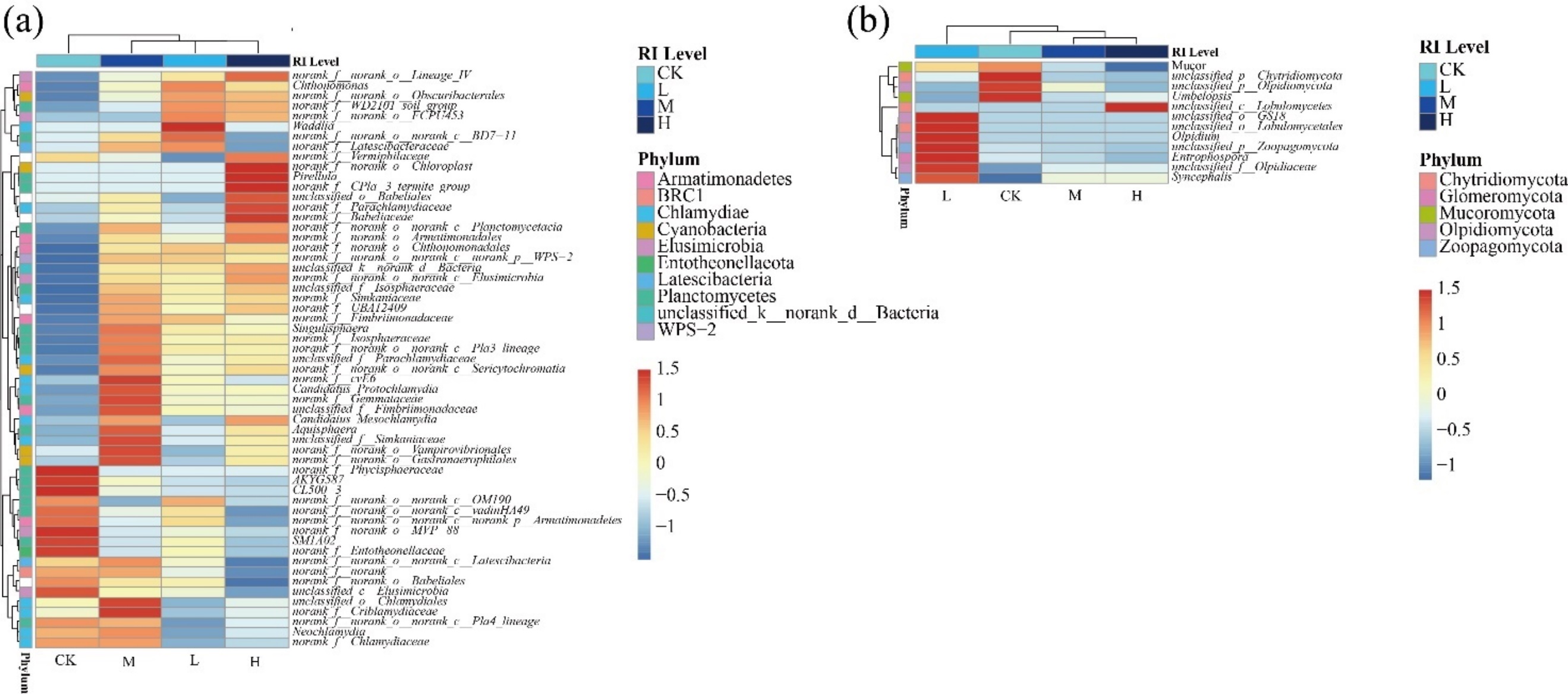


**Fig. S2. Heat map showing the relative abundance of the genera in the rare taxa for bacteria (a) and fungi (b)**


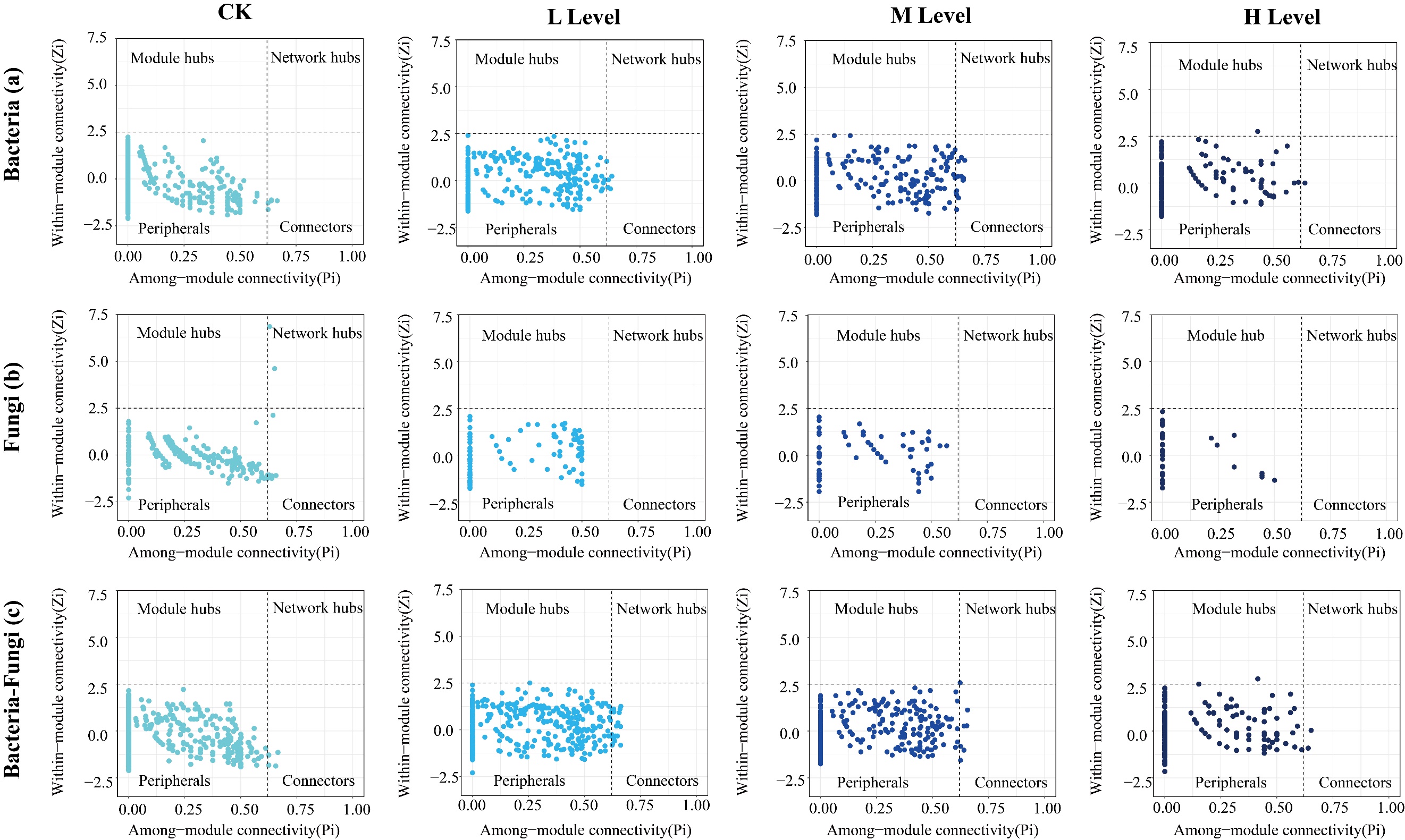


**Fig. S3.** **Identification of keystone taxa in different *RI* levels based on their topological roles in networks. (a) Bacteria; (b) Fungi; and (c) Bacteria-Fungi. Module hubs are identified as Zi > 2.5, Pi ≤ 0.62, connectors are identified as Zi ≤ 2.5, Pi > 0.62, network hubs are identified as Zi > 2.5, Pi > 0.62.**

**
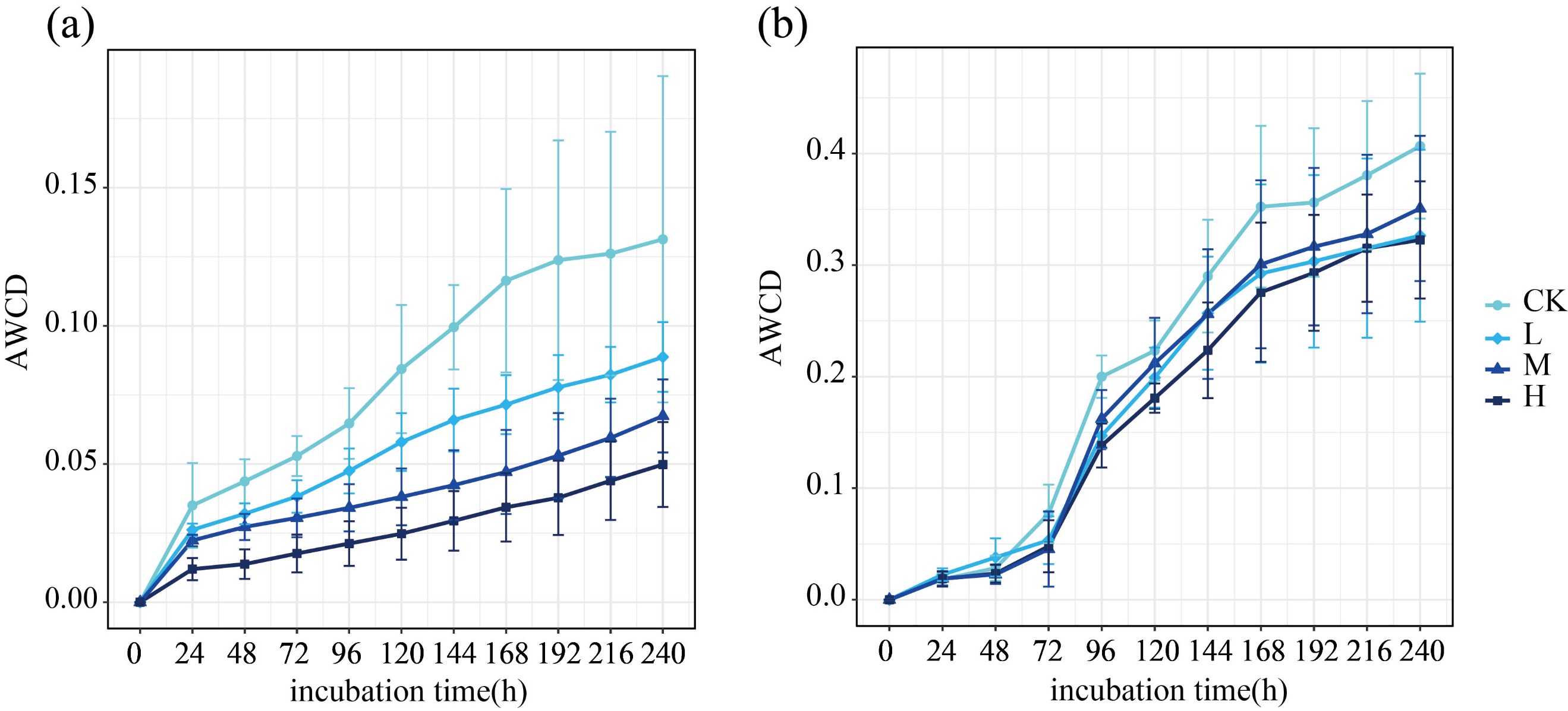
**

**Fig. S4. Average well color development (AWCD) in Biolog Ecoplate and FFplate base on 240 hours incubation.**
